# Bioinformatics and Functional Analyses Implicate Potential Roles for EOGT and *L*-fringe in Pancreatic Cancers

**DOI:** 10.3390/molecules26040882

**Published:** 2021-02-07

**Authors:** Rashu Barua, Kazuyuki Mizuno, Yuko Tashima, Mitsutaka Ogawa, Hideyuki Takeuchi, Ayumu Taguchi, Tetsuya Okajima

**Affiliations:** 1 Department of Molecular Biochemistry, Nagoya University Graduate School of Medicine, 65 Tsurumai, Showa-ku, Nagoya 466-8550, Japan; rashubarua2013@gmail.com (R.B.); tashima@med.nagoya-u.ac.jp (Y.T.); mitsutaka.ogawa@med.nagoya-u.ac.jp (M.O.); htakeuchi@med.nagoya-u.ac.jp (H.T.); 2 Division of Molecular Diagnostics, Aichi Cancer Center, 1-1 Kanokoden, Chikusa-ku, Nagoya, Aichi 464-8681, Japan; k.mizuno@aichi-cc.jp (K.M.); a.taguchi@aichi-cc.jp (A.T.); 3 Institute for Glyco-core Research (iGCORE), Integrated Glyco-Biomedical Research Center, Nagoya University, Furo-cho, Chikusa-ku, Nagoya 464-8601 Nagoya, Japan; 4 Division of Advanced Cancer Diagnostics, Nagoya University Graduate School of Medicine, 65 Tsurumai, Showa-ku, Nagoya 466-8550, Japan

**Keywords:** EOGT, LFNG, Notch, PDAC

## Abstract

Notch signaling receptors, ligands, and their downstream target genes are dysregulated in pancreatic ductal adenocarcinoma (PDAC), suggesting a role of Notch signaling in pancreatic tumor development and progression. However, dysregulation of Notch signaling by post-translational modification of Notch receptors remains poorly understood. Here, we analyzed the Notch-modifying glycosyltransferase involved in the regulation of the ligand-dependent Notch signaling pathway. Bioinformatic analysis revealed that the expression of epidermal growth factor (EGF) domain-specific *O*-linked *N*-acetylglucosamine (*EOGT*) and Lunatic fringe (*LFNG*) positively correlates with a subset of Notch signaling genes in PDAC. The lack of *EOGT* or *LFNG* expression inhibited the proliferation and migration of Panc-1 cells, as observed by the inhibition of Notch activation. *EOGT* expression is significantly increased in the basal subtype, and low expression of both *EOGT* and *LFNG* predicts better overall survival in PDAC patients. These results imply potential roles for EOGT- and LFNG-dependent Notch signaling in PDAC.

## 1. Introduction

Pancreatic cancer is one of the most fatal malignancies, which causes cancer mortality in most developed countries. It was the fourth leading cause of cancer death in 2017 in the United States, and approximately 57,000 adults were diagnosed with pancreatic cancer in 2020, which represented approximately 3% of the total cancer cases [1,2]. Generally, the 5-year survival rate of pancreatic cancer is approximately 4% [3]. More than 90% of pancreatic cancer is considered pancreatic ductal adenocarcinoma (PDAC) [4]. Typically, PDAC is diagnosed at a later stage of the disease, with highly aggressive malignancies. For this reason, patients do not receive adequate surgical treatment or conventional chemotherapy [5]. Therefore, it is necessary to determine the underlying molecular mechanism of tumor growth in PDAC to develop new therapeutic techniques that can prevent tumor progression.

The Notch signaling pathway is an evolutionarily conserved, intercellular signaling pathway involved in the cell fate decision in many biological contexts, and plays a vital role in oncogenesis [6,7]. Accordingly, Notch signaling is considered a molecular target for different malignancies [8,9]. In mouse models, both activation and inhibition of Notch signaling promote PDAC development and progression [10,11,12,13]. There are four types of Notch receptors (Notch 1–4) and five types of canonical Notch ligands: Delta-like 1 (DLL1), DLL3, DLL4, Jagged 1 (JAG1), and JAG2. The interaction of epidermal growth factor (EGF)-like repeats of Notch receptors with their ligands initiates the Notch signaling pathway [14]. Most of the EGF repeats are post-translationally modified by *O*-glycan, such as *O*-glucose (*O*-Glc), *O*-fucose (*O*-Fuc), and *O*-linked *N*-acetylglucosamine (*O*-GlcNAc) [7]. Notch signaling is a crucial factor in the progression of PDAC. It is also responsible for the EMT phenotype and cancer stem cell formation in PDAC. It has also been noted that Notch signaling components and their target genes are unregulated in invasive PDAC [15,16,17,18,19,20]. Nonetheless, dysregulated Notch signaling by the post-translational modification of Notch receptors remains poorly understood, with little research focusing on the glycosyltransferases modulating Notch activity. 

EGF domain-specific *O*-GlcNAc-transferase (EOGT) is an endoplasmic reticulum (ER)-specific enzyme, which adds an *O*-GlcNAc moiety to the extracellular domain of secreted or membrane proteins, including Notch receptors and their ligands [21,22]. Evidence suggests that the mutation of *EOGT* causes a rare congenital disease, Adams-Oliver syndrome [23,24,25]. *EOGT* is highly expressed in endothelial cells and regulates optimal vascular integrity and development by enhancing DLL ligand-mediated Notch signaling [24,26]. However, there is no evidence showing that EOGT-mediated *O*-GlcNAcylation participates in the development and progression of cancer, including PDAC. 

In addition to *EOGT*, Fringe genes are involved in the regulation of ligand-dependent Notch signaling pathway. Fringe encodes β-1,3-GlcNAc-transferase modifying *O*-Fuc on Notch receptors. In mammals, Fringe activity is mediated by three different genes (*LFNG*, *RFNG,* and *MFNG*), of which LFNG has significantly higher catalytic activity than others [27]. Unlike EOGT, LFNG enhances Delta-ligand-mediated Notch signaling but inhibits JAG1-mediated Notch signaling [28]. Though some evidence has shown that loss of *Lfng* in mice accelerated *Kras*-induced pancreatic cancer development, the roles of *LFNG* in human cancer cells are cell type-dependent and not fully understood [29]. 

To examine the contribution of *EOGT* and *LFNG* in PDAC, we conducted database analysis and functional studies in a PDAC cell line. Our study indicated critical roles for *EOGT*- and *LFNG*-dependent Notch signaling in PDAC. Furthermore, we showed—for the first time—that *EOGT* impacts cell proliferation and migration in cancer cells.

## 2. Results and Discussion

### 2.1. Contribution of EOGT and LFNG to Notch Signaling in PDAC

**Figure 1:**
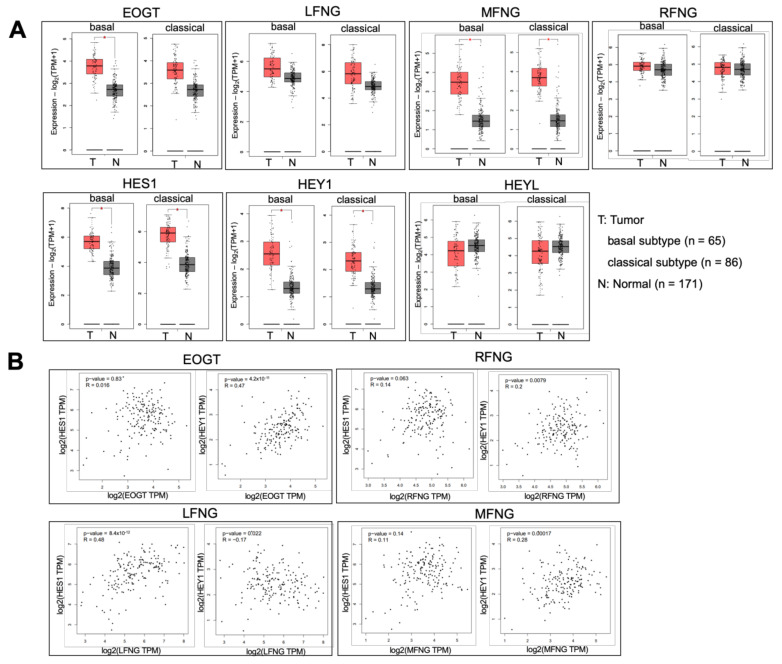
Expression of epidermal growth factor (EGF) domain-specific *O*-linked *N*-acetylglucosamine (*EOGT*) and Lunatic fringe (*LFNG*) in pancreatic ductal adenocarcinoma (PDAC) analyzed by the GEPIA2 database. (**A**) Gene expression patterns between normal and PDAC tissues analyzed using the GEPIA2 database. *p < 0.01. Red bars indicate PDAC tissues (basal or classical subtypes), and gray bars normal tissues (TCGA normal and GTEx data). (**B**) The correlation in gene expression between glycosyltransferase genes (*EOGT*, *LFNG*, *RFNG*, or *MFNG*) and Notch target genes (*HES1* or *HEY1*) was analyzed using the GEPIA2 database.

Notch target gene expression is dysregulated in PDAC [15,16,19,20]. To date, the expression levels of EOGT and FNG genes have been experimentally shown to affect both *O*-glycan structures and functions in mammals [26,28,30,31,32,33]. To investigate the expression of *EOGT* and FNG genes in PDAC, in silico analysis was performed using the GEPIA2 integrated database, which includes 179 tumor and 171 normal tissue samples. Among Notch target genes, *HES1* and *HEY1* expressions were higher in both basal and classical subtypes of PDAC. In contrast, *EOGT* expression was significantly increased in the basal subtype, which represents a more aggressive phenotype [34]. *LFNG* expression was not significantly altered (Figure 1A). However, these data did not exclude the possibility of multiple, rather than a single, glycosyltransferase(s) contributing to dysregulated Notch signaling in PDAC.

Previous studies demonstrated that the loss of EOGT decreased DLL ligand-induced Notch signaling and downstream Notch target genes [26]. Thus, we analyzed the correlation between *EOGT* and the upregulated Notch target genes (Figure 1B). Spearman correlation coefficient was used to assess monotonic correlations. Correlation analysis revealed a significant positive correlation between *EOGT* and *HEY1* (*r* = 0.47, *p* = 4.2 × 10^−11^). In contrast, the correlation analysis between *LFNG* and NOTCH target genes revealed a positive correlation between *LFNG* and *HES1* expression (*r* = 0.48, *p* = 8.4 × 10^−12^). A negligible correlation was observed for *MFNG* and *RFNG* [28]. These data suggested that the contribution of *EOGT* and *LFNG* to Notch signaling is qualitatively different, possibly through the differential impact on multiple Notch-ligand pairs, which leads to the transactivation of distinct sets of Notch signaling target genes, including *HES1* and *HEY1* [35]. Further in-depth bioinformatics analyses included Biclustering methods, will help elucidate the pathological relevance of the observed correlations in tumor progression [36,37,38,39].

### 2.2. Expression of EOGT in PDAC Cell Lines

**Figure 2:**
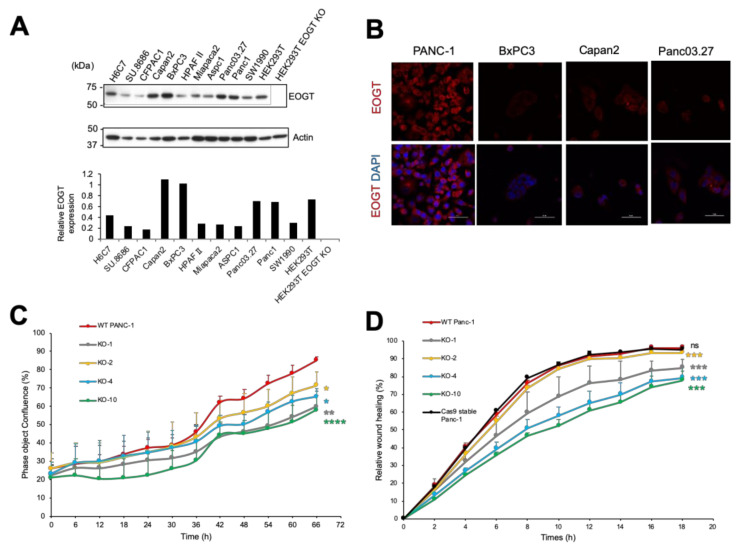
*EOGT* promotes the proliferation and migration of Panc-1 cells. (**A**) Cell lysates prepared from different pancreatic cancer cells were analyzed in parallel with cell lysates from HEK293T and HEK293T *EOGT*-KO cells. Immunoblotting was performed using anti-AER61 or beta-actin antibodies. EOGT expression level was normalized to the expression of beta-actin (below). (**B**) Detection of endogenous EOGT with EOGT-specific anti-AER61 (Red) antibody. Scale bar, 50 µm. (**C**) Wild-type (WT) and *EOGT*-KO Panc-1 cells were grown, and cell proliferation was measured by the IncuCyte ZOOM system. The data were expressed as mean ± standard deviation (SD) of triplicate samples. (**p = 0.0018 in WT vs. KO-1, *p = 0.0215 in WT vs. KO-2, *p = 0.0154 in WT vs. KO-4, and ****p < 0.0001 in WT vs. KO-10) (**D**) Cell migration assay was performed using the IncuCyte ZOOM system. At different time points, relative wound density of WT and *EOGT*-KO Panc-1 cells were measured for 18 h. The measurements are from wounds made on a monolayer of cultured cells. Each data point is expressed as mean ± SD (n = 3). ***p = 0.0009 in WT vs. KO-1, ****p = 0.0004 in WT vs. KO-2, ***p = 0.008 in WT vs. KO-4, ***p = 0.0007 in WT vs. KO-10, and *p* = 0.7568 in WT vs. Cas9-transfected control cells (Cas9 stable Panc-1).

We compared endogenous expression levels of EOGT in human PDAC cell lines to a normal pancreatic ductal cell line, H6C7. From immunoblotting data, we found that four PDAC cell lines (Panc-1, BxPC3, Panc03.27, and CAPAN-2) out of ten showed higher *EOGT* expression compared to H6C7 cells (Figure 2A). We also verified the expression of EOGT by immunostaining in the four PDAC cell lines (Figure 2B). Based on the immunoblotting and immunostaining assays, we selected Panc-1, which showed prominent EOGT expression, for functional analysis. 

### 2.3. CRISPR/CAS9-Mediated Lentiviral Knockout of EOGT in a PDAC Cell Line

To assess the role of *EOGT* in the growth of the PDAC cell line, we performed gene editing with the CRISPR/Cas9-mediated lentivirus technique in Panc-1 cells. After lentiviral transduction and blasticidin S selection, the lack of EOGT was confirmed by the anti-EOGT (AER61) antibody (Supplementary Figure S1). 

### 2.4. EOGT Knockout in Panc-1 Cells Impairs Cell Proliferation and Migration

To investigate whether the decreased Notch activity inhibits Panc-1 cell proliferation, Panc-1 cells were treated with DAPT, a γ-secretase inhibitor that blocks Notch signaling. As shown in Supplementary Figure S2, the inhibitory effect of DAPT on the proliferation of Panc-1 cells was observed in a time-dependent manner. This result suggested that decreased Notch activity can substantially reduce the cell proliferation of Panc-1. These data agree with previous reports showing a similar effect on cell proliferation by DAPT treatment [40,41]. 

To examine the effect of knockout of *EOGT* in the Panc-1 cells, we observed the proliferation between wild-type and *EOGT*-KO cells at an interval of 6 h over 66 h. *EOGT*-KO cells exhibited slow growth relative to wild-type parental control cells. This result showed that *EOGT* promotes cell proliferation in Panc-1 cells (Figure 2C).

It has been reported that inhibition of Notch1 affects cancer cell migration and invasion in vitro in pancreatic cancer [42,43]. We analyzed the cell motility of the parental wild-type and *EOGT*-KO Panc-1 by wound-healing migration assay. Analysis of the relative wound-healing density revealed that *EOGT*-KO Panc-1 showed significant decreases in re-capturing the wound portion compared to wild-type control cells (Figure 2D). Compared to the control cells, *EOGT*-KO-1, KO-2, KO-4, KO-10 clones showed a slower migration rate, presenting a depletion of wound density 13, 4, 18, and 20%, respectively, within 18 h. The differences in the migration rate among KO clones appear to be due to cancer cells’ clonal variation. According to previous studies, the doubling time of Panc-1 is 52–56 h. Therefore, the effects on the wound-healing density were predominantly due to decreased cell migration of *EOGT*-KO Panc-1 cells, but not to decreased proliferation [44,45].

### 2.5. CRISPR/Cas9-Mediated Lentiviral Knockout of LFNG in a PDAC Cell Line 

To compare the effect of EOGT and LFNG in the growth and motility of Panc-1 cells, we generated *LFNG*-KO cells by CRISPR/Cas9-mediated lentivirus transduction. The cutting efficacy was analyzed by T7 endonuclease assay. Then, by limiting dilution, single-cell clones were isolated. GFP-positive clones were selected for sequencing to confirm *LFNG* knockout in Panc-1 cells (Supplementary Figure S3). 

### 2.6. LFNG Knockout in Panc-1 Cells Impairs Cell Proliferation and Migration

**Figure 3:**
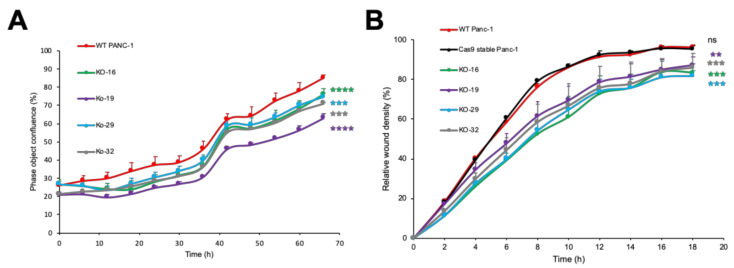
*LFNG* promotes the proliferation and migration of Panc-1 cells. (**A**) Wild-type (WT) and *LFNG*-KO Panc-1 cells were subjected to a proliferation assay with the IncuCyte ZOOM system. The data are expressed as mean ± standard deviation (SD) of triplicate samples. (****p < 0.0001 in WT vs. KO-16, ****p < 0.0001 in WT vs. KO-19, ***p = 0.0001 in WT vs. KO-29, and ****p < 0.0001 in WT vs. KO-32) (**B**) Cell migration of WT and *LFNG*-KO Panc-1 was determined by the IncuCyte ZOOM system. Each data point is expressed as mean ± SD (n = 3). ***p = 0.0006 in WT vs. KO-16, ***p = 0.0017 in WT vs. KO-19, ***p = 0.0003 in WT vs. KO-29, ***p = 0.0005 in WT vs. KO-32, and *p* = 0.7568 in WT vs. Cas9-transfected control cells (Cas9 stable Panc-1).

To evaluate the role of *LFNG* in cell proliferation, Panc-1 cells lacking *LFNG* were analyzed by the IncuCyte ZOOM system (Sartorius, Japan). The result showed that the cell proliferation of *LFNG*-KO cells was lower than that of parental control cells (Figure 3A). These data suggested that *LFNG* promotes cell proliferation of Panc-1 cells. According to previously reported data, a similar experiment was performed by shRNA-mediated knockdown of *LFNG* [29]. Although this group reported the tumor-suppressive behavior of *LFNG* in mice and pancreatic cancer cells, our findings suggested that *LFNG* promotes the proliferation activity of Panc-1 cells, which was also noted in the same study [29]. 

Then, we used the wound-healing assay to compare the motility of wild-type and *LFNG*-KO Panc-1 cells. The relative wound density revealed that the cell migration rate in controls was 10–15% faster than *LFNG*-KO Panc-1 cells (Figure 3B). These results suggest that *LFNG* promotes cell migration in a similar way to *EOGT*. 

### 2.7. Low Expression of Both EOGT and LFNG Predicts Better Overall Survival in PDAC Patients

**Figure 4:**
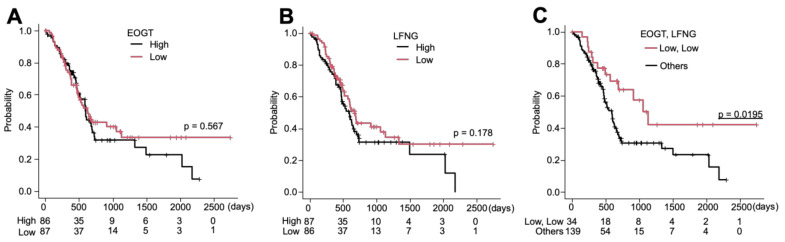
Kaplan–Meier curves showing the overall survival analysis in PDAC patients with high and low expression of *EOGT* and *LFNG*. (**A**) Association between overall survival and *EOGT* expression. (**B**) Association between overall survival and *LFNG* expression. (**C**) Lower *EOGT* and *LFNG* expression are associated with better overall survival. *p* = 0.0195, by log-rank test.

Knowing that *EOGT* and *LFNG* modulate cell proliferation and migration, the Cancer Genome Atlas (TCGA) dataset was analyzed to evaluate the clinical outcome. For overall survival, PDAC patients were classified into high and low expression groups using the median expression of genes as the cut-off value. Kaplan–Meier survival curves revealed no association between *EOGT* expression and overall survival in PDAC (Figure 4A). Additionally, no association was observed between *LFNG* expression and overall survival (Figure 4B). In contrast, lower expression of both *EOGT* and *LFNG* is associated with a better prognosis of PDAC (*p* = 0.0195). These data suggest that the dysregulation of Notch signaling mediated by high expression of either *EOGT* or *LFNG* leads to poor prognosis of PDAC.

In summary, we showed the roles of *EOGT* in cell proliferation and migration in cancer cells. In our study, loss of *EOGT* or *LFNG* resulted in a similar cellular phenotype, suggesting that both genes cooperate in the tumor properties of PDAC cells. Furthermore, the combination of lower *LFNG* and *EOGT* expression in PDAC serves as an excellent prognostic marker, implying that both Notch modifiers modulate disease progression. Given that EOGT and LFNG modulate ligand-induced Notch signaling, these results suggest that changes in the expression of these glycosyltransferases result in altered Notch activity, as indicated by the correlation between *LFNG* and *HES1*, or *EOGT* and *HEY1* expression in the PDAC database. Therefore, dysregulated Notch signaling mediated by changes in the expression of *EOGT* and *LFNG* would affect the prognosis of PDAC. Although this study implicates potential roles for *EOGT*- and *LFNG*-dependent Notch signaling in PDAC, further studies will be necessary to clarify the roles of Notch-modifying enzymes in dysregulated Notch activity and the development and progression of PDAC at the molecular level.

## 3. Materials and Methods

### 3.1. Antibodies and Reagents

The antibodies used were the following: anti-AER61 (EPR12944) (ab190693, Abcam, Japan), anti-rabbit IgG HRP-linked antibody (#7074, Cell Signaling, Japan), DyLight 549-conjugated goat anti-rabbit IgG antibody (DI-1549, Vector Laboratories, USA). The reagents used were the following: LentiX concentrator (TaKaRa, Japan), blasticidin S (Funakoshi, Japan), puromycin (MERCK, Japan), doxycycline (TaKaRa, Japan), DMSO (FUJIFILM Wako, Japan), T7 endonuclease I reaction mix (Nippon Gene, Japan). Primers specific for *EOGT* and *LFNG*, CRISPR/Cas9 sgRNA, and G-blocks were purchased from Integrated DNA Technologies (IDT, USA).

### 3.2. Cell Culture

Panc-1, LentiX-293T, HEK293T, and HEK293T *EOGT*-KO cells were cultured in Dulbecco’s modified Eagle’s medium (DMEM) supplemented with 10% fetal bovine serum (FBS). BxPC3, Panc03.27, and Capan-2 were maintained in RPMI medium supplemented with 10% FBS. 

### 3.3. Vectors

pLP1, pLP2, pVSV-G were purchased from Thermo Fisher, Japan. pLX-sgRNA (#50662), pLRG2.1 (#108098), and doxycycline-inducible pCW-Cas9 (#50661) lentivirus vectors were from Addgene, USA. 

### 3.4. Cell Growth Inhibition by DAPT

Panc-1 cells (3 × 10^4^) were seeded in a 24-well plate with 500 µL of DMEM/10% FBS. After 24 h incubation, cells were treated with 5 µM, 50 µM, and 75 µM DAPT over 69 h. Control Panc-1 cells were treated with 0.1% DMSO in the culture medium. Cell growth inhibition was measured using IncuCyte ZOOM Live-Cell Analysis System (Sartorius) [46]. Data were analyzed using phase object confluence, which quantified the cell surface area as confluence values. The images of the cells were taken every 3 h with a 4× objective.

### 3.5. Lentivirus Vector Construction

For the *EOGT* sgRNA lentivirus vector construction, *EOGT* sgRNA was designed using online software CRISPRdirect [47]. The sgRNA was synthesized as a g-block segment, which includes XhoI restriction site, U6 promoter region, sgRNA target site, chimeric sgRNA scaffold, and NheI restriction site, as shown in Supplementary Table S1. The g-block was cloned into a pLX-sgRNA lentivirus vector containing blasticidin *S*-resistance gene [48]. For selecting *LFNG* sgRNA, we used previously validated sgRNA for *LFNG* (Supplementary Table S1) [49]. The sgRNA sequence was inserted into the pLRG2.1 vector, which contains GFP as a selection marker [50]. A separate doxycycline-inducible FLAG-Cas9 expression vector (pCW-Cas9) was also used, which contains a puromycin resistance gene [48].

### 3.6. CRISPR/Cas9-Mediated Lentiviral Knockout of EOGT and LFNG Genes

The knockout experiments in Panc-1 cells were performed as described before with a slight modification [50,51]. Briefly, lentiviral plasmids are co-transfected with LP1, LP2, and VSVG virus-packaging plasmid in LentiX-293T cells using PEI MAX (Polysciences, USA). After 12h later, culture media were replaced with a high FBS containing medium (DMEM/40% FBS), which did not contain penicillin and streptomycin. After 24 and 48 h later, lentivirus supernatant was collected, centrifuged at 12,000 rpm for 5 min, and filtered using a 0.45 µm filter. After filtration, the virus was concentrated by LentiX concentrator according to the manufacturer protocol. Cas9-transfected Panc-1 cells were generated after selection with 2 µg/mL puromycin. Anti-FLAG antibody was used to verify Cas9 protein expression by immunoblotting. For generating *EOGT*-KO Panc-1 cells, the Cas9 stable Panc-1 cells were infected with lentiviral supernatant, which contains *EOGT* sgRNA. After 24 h, cells were selected with 20 µg/mL blasticidin S for 14 days, and treated with 1 µg/mL doxycycline for 1 day to induce Cas9 expression. For preparing *LFNG*-KO Panc-1 cells, a lentiviral solution was used to infect the Cas9 stable Panc-1 cells for 12 h. After 12 h, cells were treated with 1 µg/mL doxycycline for 1 day to induce Cas9 expression. The transduction efficiency was confirmed by monitoring GFP expression. Both *EOGT-* and *LFNG*-KO cells were cultured in DMEM/10% FBS medium containing penicillin and streptomycin. A single cell was isolated by limiting dilution and seeding into 96-well plates for clonal selection and further analysis. 

### 3.7. Evaluation of CRISPR/Cas9-mediated Genome Editing by T7 Endonuclease Assay and Sequencing Analysis

Genomic DNA was extracted from wild-type Panc-1, clonally selected *EOGT*-KO Panc-1, and *LFNG*-KO Panc-1 cells. Briefly, all cells were cultured in 12-well plates. After harvesting, cells were centrifuged and washed with phosphate-buffered saline (PBS). Cells were heated at 95 °C for 20 min after adding 18 µL of 50 µM NaOH for each well of 12-well plates. Then, samples were vortex briefly, added with 3 µL of 1 M Tris-HCl, pH 7.6, and centrifuged at 15,000 rpm for 6 min after vortex to collect the supernatant. The target region was amplified with sgRNA-specific primers according to the manufacturer protocol. To detect double-stranded DNA mismatches and mutations, we used T7 endonuclease according to manufacture protocol. After confirming the knockout cells, single cells were isolated by limiting dilution. For *EOGT*-KO, of the 22 single-cell clones picked out, 10 clones showed negative expression of EOGT in Western blotting screening (Supplementary Figure S1B). We selected four clones for further analysis. To select *LFNG*-KO clones, the target regions of both wild-type and knockout cells were sequenced by Sanger sequencing. Four clones were selected for *LFNG*-KO Panc-1 cells after comparing with the wild-type sequence (Supplementary Figure S3). 

### 3.8. Immunostaining

Panc-1 cells were fixed with 4% paraformaldehyde in PBS for 15 min and ice-cold methanol for 5 min. Then cells were permeabilized with 0.5% Triton-X 100 in PBS for 15 min and blocked with 5% BSA in PBS. Cells were incubated with Anti-AER61 (1:200) antibody diluted with 5% BSA/PBS overnight at 4 °C. After washing with PBS, cells were incubated with DyLight 549-conjugated goat anti-rabbit IgG antibody (1:1000) diluted with 5% BSA/PBS for 1 h at room temperature. Then, cells were counter-stained with DAPI (Southern Biotech, Birmingham, AL, USA). All the slides were examined by TiEA1R Confocal Microscopy with NIS Elements (Nikon, Japan) [52].

### 3.9. Western Blotting

Cells were lysed in a lysis buffer containing Tris-buffered saline (TBS), pH 7.6, 1 mM CaCl_2_, and 1% Nonidet P-40. Each sample was separated by 7.5% SDS-PAGE for Western blotting and transferred onto a PVDF membrane (Merck, Japan). The membrane was blocked in 3% BSA/PBST at room temperature for 1 h, followed by incubation with anti-AER61 antibody (1:10,000) diluted in 3% BSA/PBST for 2h. After washing with PBST three times, the membrane was incubated with Anti Rabbit HRP conjugated secondary antibody (1:8000) in 3% BSA/PBST at room temperature for 1 h. After washing with PBST, bands on the membrane were visualized with Immobilon Western chemiluminescent HRP substrate (Merck) and iBright FL1500 Imaging System (Thermo Fisher).

### 3.10. Cell Proliferation Assay

Cells were seeded 96-well plates at 7 × 10^3^ cells per well in DMEM medium containing 10% FBS. The increasing confluence of the cells was measured as an indicator of cell proliferation using IncuCyte ZOOM Live-Cell Analysis System. Images were taken at 6 h intervals with a 4× objective from 3 separate wells, and mean ± SD of confluence percentage was measured. 

### 3.11. Cell Migration Assay 

Cell migration (wound healing) assay was performed as reported previously [53]. Briefly, cells (2 × 10^4^) were seeded into an uncoated 96-well plate image lock plate (Sartorius). After 6–8 h later, the wound was made using 96-pin WoundMaker (Sartorius). The images of the wound were taken every 2 h for 18 h. Then, data were analyzed by calculating the relative wound density with IncuCyte ZOOM Live-Cell Analysis System.

### 3.12. Statistical Analysis and Database Analysis 

The significance of the data in functional assays was analyzed with one-way repeated measures ANOVA followed by Dunnett’s Post-hoc test to show a significant difference between groups. Significance was evaluated based on the *p*-value. Gene expression and correlation were analyzed using the GEPIA2 [54] database for pancreatic adenocarcinoma (PAAD), in which the pre-processed RNA-seq datasets and a normalization method that calculates transcripts per million (TPM) were utilized. Survival analyses were analyzed with a TCGA dataset and EZR software (version 1.52), which is a graphical user interface for R software (version 4.0.2).

## Data Availability

The data presented in this study are available in Supplementary Materials.

## Supplementary Materials

Supplementary materials are available online. Supplementary Figure S1, characterization of *EOGT*-KO Panc-1 cells; Supplementary Figure S2, the inhibitory effect of DAPT on the proliferation of Panc-1 cells; Supplementary Figure S3, sequencing data of wild-type and *LFNG*-KO Panc-1 cells; Supplementary Table S1, primers and synthetic oligonucleotides used for Crispr/Cas9-mediated genome editing.

## References

[1] Bray F., Ferlay J., Soerjomataram I., Siegel R.L., Torre L.A., Jemal A. (2018). Global cancer statistics 2018: GLOBOCAN estimates of incidence and mortality worldwide for 36 cancers in 185 countries. CA Cancer J. Clin..

[2] Siegel R.L., Miller K.D., Jemal A. (2020). Cancer statistics, 2020. CA Cancer J. Clin..

[3] Vincent A., Herman J., Schulick R., Hruban R.H., Goggins M. (2011). Pancreatic cancer. Lancet.

[4] Kleeff J., Korc M., Apte M., La Vecchia C., Johnson C.D., Biankin A.V., E Neale R., Tempero M., Tuveson D.A., Hruban R.H. (2016). Pancreatic cancer. Nat. Rev. Dis. Prim..

[5] Bliss L.A., Witkowski E.R., Yang C.J., Tseng J.F. (2014). Outcomes in operative management of pancreatic cancer. J. Surg. Oncol..

[6] Ntziachristos P., Lim J.S., Sage J., Aifantis I. (2014). From Fly Wings to Targeted Cancer Therapies: A Centennial for Notch Signaling. Cancer Cell.

[7] Tashima Y., Okajima T. (2018). Congenital diseases caused by defective *O*-glycosylation of Notch receptors. Nagoya J. Med Sci..

[8] Espinoza I., Miele L. (2013). Notch inhibitors for cancer treatment. Pharmacol. Ther..

[9] Miele L., Espinoza I., Pochampally R., Watabe K., Xing F. (2013). Notch signaling: Targeting cancer stem cells and epithelial-to-mesenchymal transition. OncoTargets Ther..

[10] Avila J.L., Kissil J.L. (2013). Notch signaling in pancreatic cancer: Oncogene or tumor suppressor?. Trends Mol. Med..

[11] Nakhai H., Siveke J.T., Klein B., Mendoza-Torres L., Mazur P.K., Algül H., Radtke F., Strobl L., Zimber-Strobl U., Schmidt G. (2008). Conditional ablation of Notch signaling in pancreatic development. Development.

[12] Hanlon L., Avila J.L., Demarest R.M., Troutman S., Allen M., Ratti F., Rustgi A.K., Stanger B.Z., Radtke F., Adsay V. (2010). Notch1 Functions as a Tumor Suppressor in a Model of K-ras–Induced Pancreatic Ductal Adenocarcinoma. Cancer Res..

[13] Agrawal N., Frederick M.J., Pickering C.R., Bettegowda C., Chang K., Li R.J., Fakhry C., Xie T.-X., Zhang J., Wang J. (2011). Exome Sequencing of Head and Neck Squamous Cell Carcinoma Reveals Inactivating Mutations in NOTCH1. Science.

[14] Kopan R., Ilagan M.X.G. (2009). The Canonical Notch Signaling Pathway: Unfolding the Activation Mechanism. Cell.

[15] Plentz R., Park J., Rhim A.D., Abravanel D., Hezel A.F., Sharma S.V., Gurumurthy S., Deshpande V., Kenific C., Settleman J. (2009). Inhibition of γ-Secretase Activity Inhibits Tumor Progression in a Mouse Model of Pancreatic Ductal Adenocarcinoma. Gastroenterology.

[16] Miyamoto Y., Maitra A., Ghosh B., Zechner U., Argani P., A Iacobuzio-Donahue C., Sriuranpong V., Iso T., Meszoely I.M., Wolfe M.S. (2003). Notch mediates TGFα-induced changes in epithelial differentiation during pancreatic tumorigenesis. Cancer Cell.

[17] Ye J., Wen J., Ning Y., Li Y. (2018). Higher notch expression implies poor survival in pancreatic ductal adenocarcinoma: A systematic review and meta-analysis. Pancreatology.

[18] Thomas M.M., Zhang Y., Mathew E., Kane K.T., Maillard I., Di Magliano M.P. (2014). Epithelial Notch signaling is a limiting step for pancreatic carcinogenesis. BMC Cancer.

[19] Song H., Wang Y., Lan H., Zhang Y. (2018). Expression of Notch receptors and their ligands in pancreatic ductal adenocarcinoma. Exp. Ther. Med..

[20] Ma J., Xia J., Miele L., Sarkar F.H., Wang Z. (2013). Notch Signaling Pathway in Pancreatic Cancer Progression. Pancreat. Disord. Ther..

[21] Matsuura A., Ito M., Sakaidani Y., Kondo T., Murakami K., Furukawa K., Nadano D., Matsuda T., Okajima T. (2008). O-LinkedN-Acetylglucosamine Is Present on the Extracellular Domain of Notch Receptors. J. Biol. Chem..

[22] Tashima Y., Stanley P. (2014). Antibodies That Detect O-Linked β-d-N-Acetylglucosamine on the Extracellular Domain of Cell Surface Glycoproteins. J. Biol. Chem..

[23] Cohen I., Silberstein E., Perez Y., Landau D., Elbedour K., Langer Y., Kadir R., Volodarsky M., Sivan S., Narkis G. (2013). Autosomal recessive Adams–Oliver syndrome caused by homozygous mutation in EOGT, encoding an EGF domain-specific O-GlcNAc transferase. Eur. J. Hum. Genet..

[24] Ogawa M., Okajima T. (2019). Structure and function of extracellular O-GlcNAc. Curr. Opin. Struct. Biol..

[25] Hassed S., Li S., Mulvihill J., Aston C., Palmer S. (2017). Adams-Oliver syndrome review of the literature: Refining the diagnostic phenotype. Am. J. Med Genet. Part A.

[26] Sawaguchi S., Varshney S., Ogawa M., Sakaidani Y., Yagi H., Takeshita K., Murohara T., Kato K., Sundaram S., Stanley P. (2017). O-GlcNAc on NOTCH1 EGF repeats regulates ligand-induced Notch signaling and vascular development in mammals. eLife.

[27] Dunwoodie S.L. (2009). Mutation of the fucose-specific β1,3 N-acetylglucosaminyltransferase LFNG results in abnormal formation of the spine. Biochim. Biophys. Acta BBA Mol. Basis Dis..

[28] Kakuda S., Lopilato R.K., Ito A., Haltiwanger R.S. (2020). Canonical Notch ligands and Fringes have distinct effects on NOTCH1 and NOTCH2. J. Biol. Chem..

[29] Zhang S., Chung W.-C., Xu K. (2016). Lunatic Fringe is a potent tumor suppressor in Kras-initiated pancreatic cancer. Oncogene.

[30] Ogawa M., Senoo Y., Ikeda K., Takeuchi H., Okajima T. (2018). Structural Divergence in O-GlcNAc Glycans Displayed on Epidermal Growth Factor-like Repeats of Mammalian Notch1. Molecules.

[31] Varshney S., Stanley P. (2018). Multiple roles for O-glycans in Notch signalling. FEBS Lett..

[32] Kakuda S., Haltiwanger R.S. (2017). Deciphering the Fringe-Mediated Notch Code: Identification of Activating and Inhibiting Sites Allowing Discrimination between Ligands. Dev. Cell.

[33] Haltom A.R., Jafar-Nejad H. (2015). The multiple roles of epidermal growth factor repeatO-glycans in animal development. Glycobiology.

[34] Juiz N., Elkaoutari A., Bigonnet M., Gayet O., Roques J., Nicolle R., Iovanna J.L., Dusetti N. (2020). Basal-like and classical cells coexist in pancreatic cancer revealed by single-cell analysis on biopsy-derived pancreatic cancer organoids from the classical subtype. FASEB J..

[35] Nandagopal N., Santat L.A., Lebon L., Sprinzak D., Bronner M.E., Elowitz M.B. (2018). Dynamic Ligand Discrimination in the Notch Signaling Pathway. Cell.

[36] Lopez-Fernandez A., Rodriguez-Baena D., Gomez-Vela F., Divina F., Garcia-Torres M. (2021). A multi-GPU biclustering algorithm for binary datasets. J. Parallel Distrib. Comput..

[37] Orzechowski P., Sipper M., Huang X., Moore J.H. (2018). EBIC: An evolutionary-based parallel biclustering algorithm for pattern discovery. Bioinformatics.

[38] Xie J., Ma A., Zhang Y., Liu B., Cao S., Wang C., Xu J., Zhang C., Ma Q. (2020). QUBIC2: A novel and robust biclustering algorithm for analyses and interpretation of large-scale RNA-Seq data. Bioinformatics.

[39] Bhattacharya A., Cui Y. (2017). A GPU-accelerated algorithm for biclustering analysis and detection of condition-dependent coexpression network modules. Sci. Rep..

[40] Du X., Cheng Z., Li Y., Zhou Z.-G., Yang L., Zhang M.-M. (2013). Suppressive effects of gamma-secretase inhibitor DAPT on the proliferation of pancreatic cancer cells. Sichuan da xue xue bao. Yi xue ban J. Sichuan Univ. Med Sci. Ed..

[41] Harbuzariu A., Rampoldi A., Daley-Brown D.S., Candelaria P., Harmon T.L., Lipsey C.C., Beech D.J., Quarshie A., Ilies G.O., Gonzalez R.R. (2016). Leptin-Notch signaling axis is involved in pancreatic cancer progression. Oncotarget.

[42] Hu Y., Su H., Ling C., Guoliang Q., Cheng L., Qin R., Qing G., Liu H. (2015). The NOTCH Ligand JAGGED2 Promotes Pancreatic Cancer Metastasis Independent of NOTCH Signaling Activation. Mol. Cancer Ther..

[43] Chen D., Wang H., Bao Y., Xie K. (2018). Notch signaling molecule is involved in the invasion of MiaPaCa2 cells induced by CoCl2 via regulating epithelial‑mesenchymal transition. Mol. Med. Rep..

[44] Lieber M., Mazzetta J., Nelson-Rees W., Kaplan M., Todaro G. (1975). Establishment of a continuous tumor-cell line (PANC-1) from a human carcinoma of the exocrine pancreas. Int. J. Cancer.

[45] Watanabe M. (2012). Metabolic Profiling Comparison of Human Pancreatic Ductal Epithelial Cells and Three Pancreatic Cancer Cell Lines using NMR Based Metabonomics. J. Mol. Biomarkers Diagn..

[46] Single A., Beetham H., Telford B.J., Guilford P., Chen A. (2015). A Comparison of Real-Time and Endpoint Cell Viability Assays for Improved Synthetic Lethal Drug Validation. J. Biomol. Screen..

[47] Naito Y., Hino K., Bono H., Ui-Tei K. (2015). CRISPRdirect: Software for designing CRISPR/Cas guide RNA with reduced off-target sites. Bioinformatics.

[48] Wang T., Wei J.J., Sabatini D.M., Lander E.S. (2014). Genetic Screens in Human Cells Using the CRISPR-Cas9 System. Science.

[49] Narimatsu Y., Joshi H.J., Yang Z., Gomes C., Chen Y.-H., Lorenzetti F.C., Furukawa S., Schjoldager K.T., Hansen L., Clausen H. (2018). A validated gRNA library for CRISPR/Cas9 targeting of the human glycosyltransferase genome. Glycobiology.

[50] Giuliano C.J., Lin A., Girish V., Sheltzer J.M. (2019). Generating Single Cell–Derived Knockout Clones in Mammalian Cells with CRISPR/Cas9. Curr. Protoc. Mol. Biol..

[51] Abrahimi P., Chang W.G., Kluger M.S., Qyang Y., Tellides G., Saltzman W.M., Pober J.S. (2015). Efficient Gene Disruption in Cultured Primary Human Endothelial Cells by CRISPR/Cas9. Circ. Res..

[52] Alam S.M.D., Tsukamoto Y., Ogawa M., Senoo Y., Ikeda K., Tashima Y., Takeuchi H., Okajima T. (2020). N-Glycans on EGF domain-specific O-GlcNAc transferase (EOGT) facilitate EOGT maturation and peripheral endoplasmic reticulum localization. J. Biol. Chem..

[53] Libério M.S., Sadowski M.C., Soekmadji C., Davis R.A., Nelson C.C. (2014). Differential Effects of Tissue Culture Coating Substrates on Prostate Cancer Cell Adherence, Morphology and Behavior. PLOS ONE.

[54] Tang Z., Kang B., Li C., Chen T., Zhang Z. (2019). GEPIA2: An enhanced web server for large-scale expression profiling and interactive analysis. Nucleic Acids Res..

