# Supplementary material for: Bioinformatics and Functional Analyses Implicate Potential Roles for EOGT and L-fringe in Pancreatic Cancers
Source: Molecules. 2021 Feb 7;26(4):882. doi: 10.3390/molecules26040882 (PMC7915272; doi:10.3390/molecules26040882)
Supplement: Supplementary file 1 [file molecules-26-00882-s001.pdf]

Supplementary Materials

Bioinformatics and Functional Analyses Implicate Potential Roles for EOGT and *L*-fringe in Pancreatic Cancers

Rashu Barua[1], Kazuyuki Mizuno[2], Yuko Tashima[1,4], Mitsutaka Ogawa[1,4], Hideyuki Takeuchi[1,4], Ayumu Taguchi[1,3] and Tetsuya Okajima[1,4*]

[1] Department of Molecular Biochemistry, Nagoya University Graduate School of Medicine, 65 Tsurumai, Showa-ku, Nagoya 466–8550, Japan; rashubarua2013@gmail.com (R.B.); tashima@med.nagoya-u.ac.jp (Y.T.); mitsutaka.ogawa@med.nagoya-u.ac.jp (M.O.); htakeuchi@med.nagoya-u.ac.jp (H.T.);
[2] Division of Molecular Diagnostics, Aichi Cancer Center, 1–1 Kanokoden, Chikusa-ku, Nagoya, Aichi 464–8681, Japan; k.mizuno@aichi-cc.jp (K.M.); a.taguchi@aichi-cc.jp (A.T.)
[3] Institute for Glyco-core Research (iGCORE), Integrated Glyco-Biomedical Research Center, Nagoya University, Furo-cho, Chikusa-ku, Nagoya 464–8601 Nagoya, Japan
[4] Division of Advanced Cancer Diagnostics, Nagoya University Graduate School of Medicine, 65 Tsurumai, Showa-ku, Nagoya 466–8550, Japan
* Correspondence: tokajima@med.nagoya-u.ac.jp; Tel.: +81-52-744-2068; Fax: +81-52-744-2069

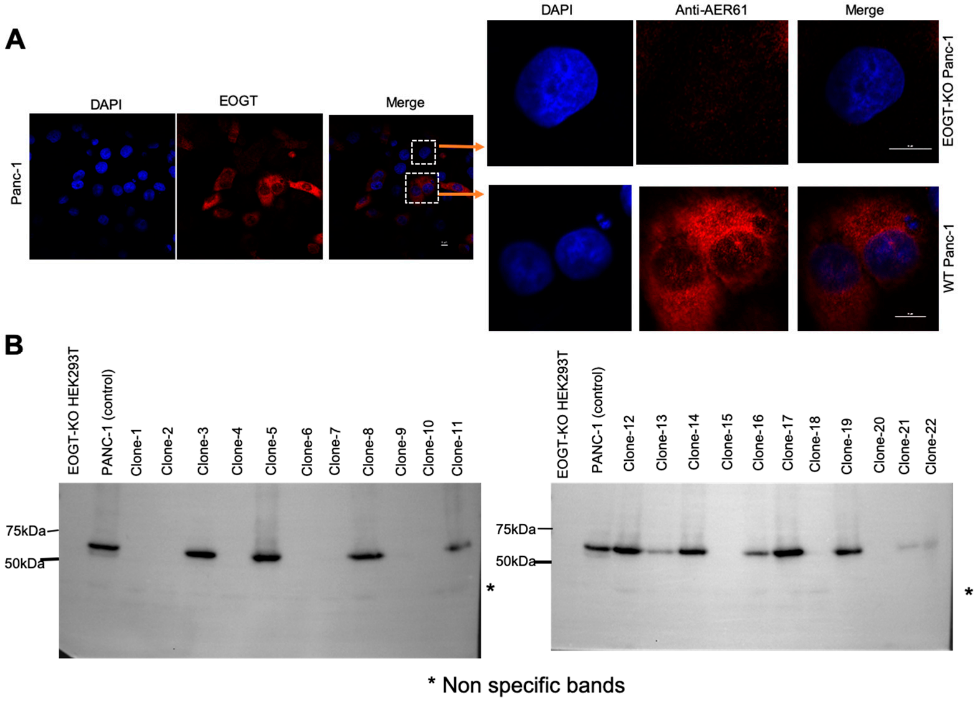

Figure S1. Characterization of *EOGT*-KO Panc-1 cells. (**A**) Confocal microscopy was performed to detect endogenous EOGT in a mixed population of wild-type (WT) and *EOGT*-KO Panc-1 cells by anti-AER61 (EOGT) antibody. Scale bar, 10 μm. (**B**) Detection of endogenous EOGT in the cell lysates of WT or *EOGT*-KO Panc-1 cells using anti-AER61 (EOGT). *EOGT*-KO HEK293T cells were used as a negative control.

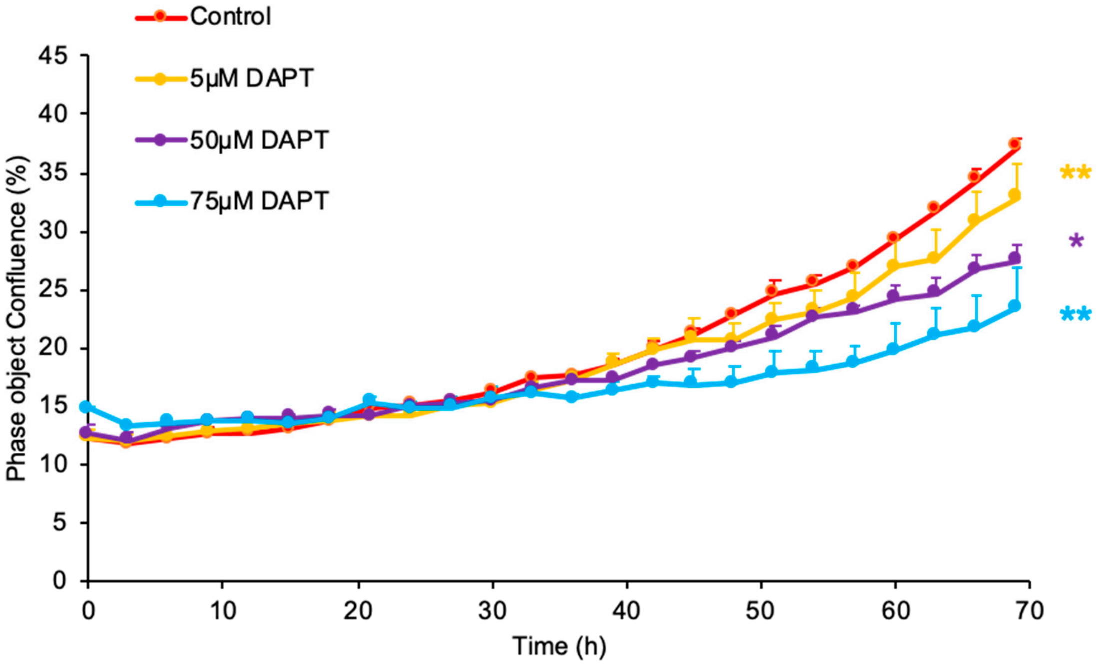

Figure S2. The inhibitory effect of DAPT on the proliferation of Panc-1 cells. Cell proliferation was monitored by the IncuCyte ZOOM system. Panc-1 cells were treated with 5, 50, or 75 μM DAPT for 69 h. Each data was expressed as means ± SD (n = 3). (***p* = 0.003 in Control vs. 5 μM DAPT, **p* = 0.0137 in Control vs. 50 μM DAPT, and ***p* = 0.0069 in Control vs. 75 μM DAPT).

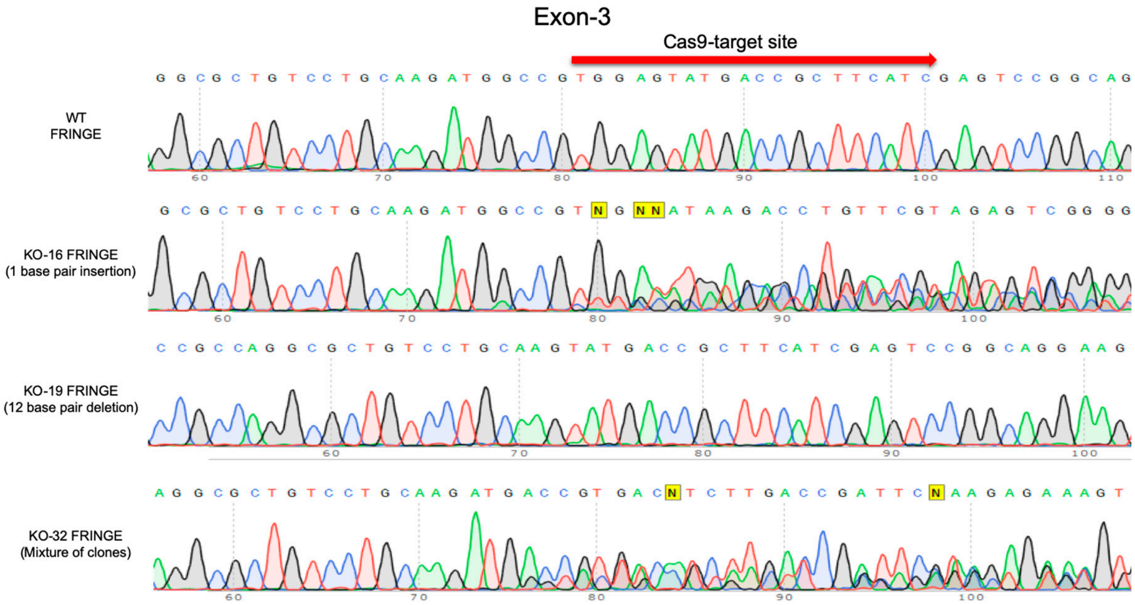

Figure S3. Sequencing data of wild-type and *LFNG*-KO Panc-1 cells. The sgRNA sequence (TGGAGTATGACCGCTTCATC) in exon 3 is underlined.

Table S1. Primers and synthetic oligonucleotides used for Crispr/Cas9-mediated genome editing.

***LFNG* sgRNA sequence (TGGAGTATGACCGCTTCATC)**
Forward primer: TGGAGTATGACCGCTTCATC
Reverse primer: GATGAAGCGGTCATACTCCA

G-block for pLX-sgRNA for EOGT (sgRNA sequence underlined)
GAGATCCACTTTGGCGCCGGCTCGAGTGTACAAAAAAGCAGGCTTTAAAGGAACCAATTC
AGTCGACTGGATCCGGTACCAAGGTCGGGCAGGAAGAGGGCCTATTTCCCATGATTCCTTC
ATATTTGCATATACGATACAAGGCTGTTAGAGAGATAATTAGAATTAATTTGACTGTAAAC
ACAAAGATATTAGTACAAAATACGTGACGTAGAAAGTAATAATTTCTTGGGTAGTTTGCAG
TTTTAAAATTATGTTTTAAAATGGACTATCATATGCTTACCGTAACTTGAAAGTATTTCGATT
TCTTGGCTTTATATATCTTGTGGAAAGGACGAAACACC<u>GGAATGCTGTGAGTATTAGG</u>GTTT
TAGAGCTAGAAATAGCAAGTTAAAATAAGGCTAGTCCGTTATCAACTTGAAAAAGTGGCA
CCGAGTCGGTGCTTTTTTTCTAGACCCAGCTTTCTTGTACAAAGTTGGCATTAGCTAGCGCTA
ACCGGTGG.
